# Binding of Pentagalloyl Glucose to Aortic Wall Proteins: Insights from Peptide Mapping and Simulated Docking Studies

**DOI:** 10.3390/bioengineering10080936

**Published:** 2023-08-07

**Authors:** Dan Simionescu, Nishanth Tharayil, Elizabeth Leonard, Wenda Carlyle, Alex Schwarz, Kelvin Ning, Christopher Carsten, Juan Carlos Carrillo Garcia, Alexander Carter, Collin Owens, Agneta Simionescu

**Affiliations:** 1 Biocompatibility and Tissue Regeneration Laboratory, Department of Bioengineering, Clemson University, Clemson, SC 29634, USA; juancac@clemson.edu (J.C.C.G.); adc3@clemson.edu (A.C.); 2 Multi-User Analytical Lab (MUAL) & Metabolomic Core, Clemson University, Clemson, SC 29634, USA; ntharay@clemson.edu (N.T.); eleona2@g.clemson.edu (E.L.); 3 Nectero Medical Inc., Mesa, AZ 85281, USA; wcarlyle@necteromedical.com (W.C.); aschwarz@necteromedical.com (A.S.); kning@necteromedical.com (K.N.); 4 PRISMA Health, Greenville, SC 29640, USA; chris.carsten@prismahealth.org; 5 Tissue Engineering Laboratory, Department of Bioengineering, Clemson University, Clemson, SC 29634, USA; crowens@clemson.edu (C.O.); agneta@clemson.edu (A.S.)

**Keywords:** aneurysms, polyphenols, diffusion, stabilization, docking simulations, elastin, collagen, elastin-associated microfibrillar proteins

## Abstract

Pentagalloyl glucose (PGG) is currently being investigated as a non-surgical treatment for abdominal aortic aneurysms (AAAs); however, the molecular mechanisms of action of PGG on the AAA matrix components and the intra-luminal thrombus (ILT) still need to be better understood. To assess these interactions, we utilized peptide fingerprinting and molecular docking simulations to predict the binding of PGG to vascular proteins in normal and aneurysmal aorta, including matrix metalloproteinases (MMPs), cytokines, and fibrin. We performed PGG diffusion studies in pure fibrin gels and human ILT samples. PGG was predicted to bind with high affinity to most vascular proteins, the active sites of MMPs, and several cytokines known to be present in AAAs. Finally, despite potential binding to fibrin, PGG was shown to diffuse readily through thrombus at physiologic pressures. In conclusion, PGG can bind to all the normal and aneurysmal aorta protein components with high affinity, potentially protecting the tissue from degradation and exerting anti-inflammatory activities. Diffusion studies showed that thrombus presence in AAAs is not a barrier to endovascular treatment. Together, these results provide a deeper understanding of the clinical potential of PGG as a non-surgical treatment of AAAs.

## 1. Introduction

1,2,3,4,6 Pentagalloyl glucose (PGG), as well as its parent compound, tannic acid (decagalloyl glucose), were first introduced in the early 1970s as efficient mordants (i.e., fixation agents that bind to tissue components and enhance further binding of metal ions) during tissue preparation for transmission electron microscopy (TEM) [1,2]. Notably, Kajikawa et al. showed that elastin could be visualized at the TEM level using a tannic acid method [3], inferring that tannic acid has a high affinity towards elastin. Since then, many papers have been published utilizing this method of highlighting elastic fibers for TEM [3,4,5]. While searching for alternative fixatives for the preparation of bioprosthetic heart valves, our group showed that tannic acid binds to pure elastin and vascular elastic fibers, protecting elastin from degradation by elastase [6]. In a previous paper, we showed that tannic-acid-mediated elastin stabilization also protects elastin from calcification in vivo [7]. In 2006, we determined that the polyphenolic groups are responsible for the stabilization properties and that PGG, resulting from tannic acid hydrolysis, is equally effective as an elastin stabilizer and more stable than tannic acid [8]. PGG has found potential applications in cardiovascular medicine exhibiting excellent safety profiles [9,10]. In 2007, using periadventitial treatment of rodent abdominal aortic aneurysms (AAAs), our group published the first in vivo evidence that the delivery of PGG to aneurysmal aorta inhibits elastin degradation and attenuates aneurysmal expansion [11]. Lindholt et al. then showed that intraluminal infusion of PGG could impair AAA development in an elastase-induced aneurysm swine model [12], and Schack et al. confirmed the beneficial effect of intraluminal PGG in rodents [13]. In 2020, Simionescu et al. reported that PGG diffuses rapidly through arterial tissues and that a single 3 min intraluminal delivery of PGG to the aneurysmal aorta was sufficient to reduce AAA expansion in a swine model [14]. The use of PGG, delivered endovascularly, to slow the growth of AAAs has now moved to first-in-human (FIH) clinical trials (https://clinicaltrials.gov/ct2/show/NCT05133492 (accessed on 5 August 2023)).

Despite extensive preclinical experimentation, the molecular mechanisms of the action of PGG on the aneurysmal wall still need to be better understood. The studies described herein were undertaken to identify molecular interactions between PGG and individual components of the aneurysmal aorta, including extracellular matrix components, cytokines, and the intra-luminal thrombus. A short description of these target elements follows.

### 1.1. Extracellular Matrix (ECM) in the Normal Aorta

**Figure 1:**
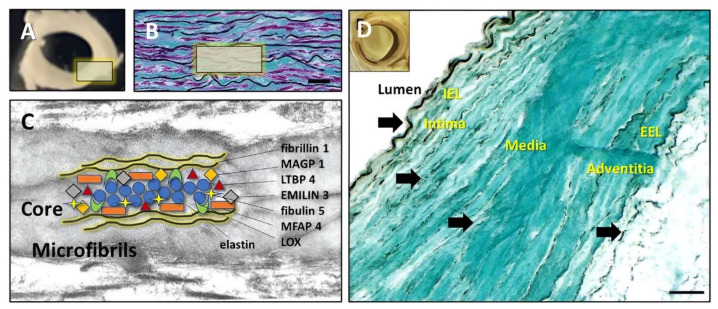
Hierarchical structure and composition of aortic elastic fibers and PGG binding patterns. (**A**) Representative cross-section through a swine abdominal artery (*n* = 3). (**B**) Thin section of swine aorta stained histologically for elastin (black), collagen (blue), and cells (purple). (**C**) TEM of a typical elastic fiber composed of an amorphous core surrounded by and interspersed with microfibrils. The molecular composition of an elastic fiber includes elastin, coacervated together with MAGP, LTBP, emilin, fibulin, MFAP, and LOX and surrounded by a fibrillin sheath. (**D**) Binding of PGG to aortic components as highlighted by iron chloride staining. Insert—macroscopic image of an en bloc FeCl_3_-stained, PGG-treated swine aorta showing intense luminal stain. Representative cryosections made from the same sample as in the insert, counterstained with Light Green, showing tissue-bound PGG in black and other matrix components in green. IEL—internal elastic lamina; EEL—external elastic lamina. Arrows point to potential PGG binding sites including fine elastic fibers in the media. Bars in (**B**,**D**) are 100 µm.

Large arteries comprise about 50% of elastic fibers, with the remainder comprising collagen and cells (Figure 1). Zooming into the ultrastructural and molecular composition, elastic fibers are complex structures composed of a dense core of amorphous, crosslinked elastin (ELN), and multiple elastin-associated microfibrillar (EAMF) proteins (Figure 1). The basis of this complexity resides in the multi-step process of elastic fiber formation, which starts with the extra-cellular secretion of the tropoelastin monomers, which coacervate into spherical aggregates on fibrillin templates that are stabilized by proteoglycans and fibulins. For a more detailed discussion of vascular extracellular matrix organization see the excellent reviews by Heinz et al. [15,16]. The elastin is cross-linked by the action of lysyl oxidase (LOX) which remains within the fiber [17]. The structure matures by acquiring other EAMF proteins such as microfibril-associated glycoprotein (MAGP), latent-transforming growth factor beta-binding protein (LTBP), microfibril-associated glycoprotein (MFAP), and elastin-microfbril-interface-located-protein (EMILIN) (Figure 1) [17,18,19]. When PGG is infused intraluminally into the abdominal aorta for 3 min or more, it diffuses rapidly through the tissue and binds to the internal elastic lamina [14]. With saturating concentrations, PGG binds the medial elastic fibers, external elastic lamina, and continues to diffuse swiftly through the vessel wall, reaching the adventitial fibers (Figure 1). The staining is most obvious when PGG is bound to multiple proteins that are coalesced into dense, thick structures such as elastic lamina. In areas of the vessel wall where structural proteins are present in thinner fibers, PGG may still be bound, but cannot be as easily visualized. Very fine fibers are highlighted in the media in Figure 1 (arrows). Within the adventitia, thicker collagen bundles may similarly provide a target for PGG binding that is readily apparent because of the mass of these fibers. Due to the complex nature of the ECM in the aorta, we decided to investigate the molecular interactions of PGG with each of these components.

### 1.2. Aneurysmal Aorta Components

An AAA is a chronic disorder characterized by the weakening and progressive dilatation of the aorta with an increasing risk of rupture. The pathogenesis of AAA is associated with cell death, localized inflammation, leucocyte infiltration, and degradation of the aortic matrix elastin and collagen fibers by matrix metalloproteinases (MMPs). Many cytokines are present in an AAA throughout its development and progression [20,21]. Among these, interleukin 1 (IL-1), IL-6 [22], IL-8, IL-10, tissue necrosis factor (TNF)-alpha, chemokine (c-c motif) ligand (CCL)-5, monocyte chemoattractant protein (MCP) 1 were found to be associated with experimentally induced AAAs, and in human AAA tissue extracts [23]. MMPs have been implicated in the pathogenesis of AAAs for decades [24], specifically focusing on MMP 2, 9 and 12. Inhibition of MMPs has been an obvious target in experimental animal models of AAAs [25]; however, in a recent clinical trial, aneurysm growth was not inhibited by systemic administration of doxycycline, an MMP inhibitor, although interestingly, the use of systemic doxycycline did inhibit local MMP activity in the AAA wall [26].

### 1.3. Intraluminal Thrombus in AAA

About 70–80% of AAA patients have an intraluminal thrombus (ILT), which generally is eccentric and does not impede blood flow [27,28]. Typically, the ILT is around 8–9 cm long, has a volume of about 90 mL, and is positioned in the anterior portion of the AAA sac [29,30]. The ILT has been considered a bystander to AAA. Recent research, however, has highlighted the role of ILT in AAA development and in the progression of endoleaks after endovascular aneurysm repair (EVAR) of abdominal aortic aneurysms [27,29]. Moreover, drug diffusion through the ILT becomes important when agents targeting the aneurysmal wall are delivered intraluminally. The transport of macromolecules through ILT likely occurs from the lumen towards the abluminal side through a system of channels or canaliculi [30]. Most channels are between 5–36 µm^2^ in area, large enough for macromolecules to diffuse through [30]. Toluidine Blue diffusion measurements through human ILT revealed a 0.91 +/− 0.54 mm^4^/N permeability, three orders of magnitude higher than that of the aortic wall or that of articular cartilage [30]. To our knowledge, research has yet to be conducted to assess the diffusivity of PGG through ILT and the molecular interactions of PGG with EAMF, MMPs, and cytokines involved in AAA.

Our working hypothesis is that PGG can bind to some of the main components of the vascular wall, including elastin, EAMF, proteoglycans, and collagen, thus accounting for the observed tissue stabilization effect of PGG. We also hypothesize that PGG could bind to MMPs and cytokines in the aneurysmal aorta as a secondary means to impact on AAA development and progression. Finally, we sought to understand whether patient ILT poses a barrier to PGG diffusion at physiologic pressures. To understand these interactions, we performed three complementary studies.

First, we exposed purified human aortic elastin to a solution containing PGG for 3 min, a duration sufficient for tissue saturation based on bench testing and preclinical studies [14]. We then evaluated the effects of PGG on elastin structure via tandem liquid chromatography mass spectrometry (LC-MS/MS) and peptide fingerprinting after elastase digestion compared to appropriate controls.

In the second experiment, we used molecular docking software to predict the most probable binding sites and calculate the affinity of PGG to the elastin protein molecule, to naturally occurring EAMF proteins, collagens, and proteoglycans, as well as to other major elements found in the AAA environment such as MMPs and cytokines.

Finally, we evaluated PGG binding to fibrin via molecular simulations and performed PGG diffusion studies in pure fibrin gels and human ILT samples.

## 2. Materials and Methods

Purified human aortic elastin (SH476) and human neutrophil elastase (HNE) (SE563) were obtained from Elastin Products Company (Owensville, MO, USA). The elastin preparation consists of pure elastin fibers, lacking any associated fiber proteins. A formulation of 3.0 mg/mL PGG concentration in buffer and contrast material (244 mg/mL iopamidol) consistent with that used in a recent human FIH clinical trial as well as vehicle controls was provided by Nectero Medical Inc., Tempe, AZ, USA. The PGG solution was neutralized with sodium bicarbonate (6.6 mL of a 4.2% solution added to 100 mL solution) before use; final pH of all solutions was 7.1. As controls we used a buffer solution without iopamidol, neutralized to pH 7.1 before use (labeled BUF), and a buffered contrast solution (without PGG), also neutralized before use (labeled CNT). All other materials and chemicals were of the highest purity available.

### 2.1. Treatment of Pure Elastin with PGG and Sample Preparation for Peptide Fingerprinting

Human aortic elastin samples, at 2 mg each, were randomly divided into three groups (*n* = 3 per group). Group 1 (labeled CNT) was treated with the neutralized vehicle control buffer with contrast material, Group 2 was treated with a neutralized vehicle control buffer without contrast material (BUF), and Group 3 was treated with buffered, neutralized 3.0 mg/mL PGG in vehicle with contrast material. Dry elastin samples were transferred to 2 mL tubes, and 1 mL solution was added to the elastin samples; the tubes were vortexed twice for 5 s and placed in a water bath at 37 °C for 3 min. The samples were centrifuged at 12,000 rpm for 5 min, and the supernatant was aspirated with a transfer pipette. The samples were rinsed twice with 1 mL saline, vortexed, centrifuged again, and the supernatant aspirated. For enzymatic digestion, human neutrophil elastase (HNE) was prepared at a concentration of 43 U/mL in phosphate-buffered saline (PBS) with 5 mM CaCl2. One (1) ml HNE solution was added to each elastin sample which were then stirred with a Teflon-coated micro magnetic stirrer at 37 °C for 20 h. To finalize digestion, more enzyme (0.5 mL of the same HNE solution) was added to each sample at 37 °C for another 20 h. Samples were centrifuged at 12,000 rpm for 5 min and filtered through a 10 k MW filter (Centricon Ultracel YM 10, 0.5 mL). The insoluble fibers, undigested elastin, and elastase remained on the filter while the filtrate contained the digested elastin peptides. Peptides were quantified on a Nanodrop machine at 205 nm and all samples were stored at −20 °C. Most samples contained around 1.6–1.8 mg peptides/mL (data not shown). Samples were transported frozen to the Multi-user Analytical Lab (MUAL) and Metabolomic Core on the Clemson campus for analysis.

### 2.2. Peptide Fingerprinting

Elastase-digested samples were prepared as described above and analyzed using LC-MS/MS. For better separation and resolution of peptide fragments, samples were first run through a C-18 spin column (Pierce 89870). Samples were then dried and reconstituted in 0.1% formic acid. Retention time calibrants were added (Pierce 88321) and samples were analyzed on a nano-LC-MS/MS system for peptide profiling using a 120 min gradient. Solvent A was 0.1% formic acid, and solvent B was 80% acetonitrile + 0.1% formic acid. The column was PepMapRSLC C18 (2 μm, 100 A, 75 μm, 50 cm). Injections were 1 µL with duplicate injections per sample, using *n* = 3 injections per group. Blanks were injected between each sample. Peptide fingerprinting yielded accurate mass and retention time of elastin peptides, and relative abundance was calculated based on the areas of each peak using the canonical human elastin (ELN) amino acid sequence (Entrez GeneID2006, UniProt P15502). Data were processed using Proteome Discoverer 2.4 and Skyline software. A comparative analysis between the three groups (BUF, CNT, PGG) was performed using the heat map function, looking at the number of peptides and the abundance of peptides in each group.

### 2.3. Molecular Docking

Docking is an in silico method used to predict the binding modes of a ligand to a target. This is a standard drug discovery pipeline for FDA-approved drugs, enzyme inhibitors, and others. The ligand is typically a small molecule; the target is a protein, an enzyme or its catalytic site, DNA sequences, etc. Molecular docking in silico can help reduce in vitro screening efforts. The main steps in docking include (1) preparing the ligand, (2) preparing the target, (3) defining the 3D reaction volume (box) within the target, (4) activating the molecular simulation software, and (5) analyzing results. There are multiple software packages available, with AutodockVina being the most popular. Most of these require additional software packages and Unix coding and a requirement for advanced knowledge. Recently, however, web interfaces incorporating all the necessary software into easy-to-use platforms have been developed. We utilized SeamDock, developed by the University of Paris, which integrates all required software into a user-friendly cloud platform [31].

The PGG structure was downloaded from Pubchem (CID 65238) in structured data file (sdf) format. The target proteins were obtained as protein data bank (pdb) files from the Research Collaboratory for Structural Bioinformatics (RCSB) data bank (RCSB.org), UniProt (uniport.org), or AlphaFold (alphafold.ebi.ac.uk) [32]. Table 1 summarizes the target proteins and their ID codes used in this docking study. To perform the docking, the SeamDock program was launched (https://bioserv.rpbs.univ-paris-diderot.fr/services/SeamDock/ (accessed on 10 April 2023)), the ligand (PGG) sdf structure and the target protein (receptor) pdb file were uploaded into the appropriate sites, and, after the software had prepared the two reactants, the interaction box coordinates were set to cover the catalytic sites for enzymes, or the entire protein molecule for most proteins, except elastin. Since elastin protein is a very large structure (786 amino acids), we performed 6 separate docking simulations using different ligand-target interaction boxes, thus systematically covering the entire molecule. The docking parameters were set at Vina software, mode 2, energy range 12, exhaustiveness 8, and the docking program was launched. After the docking process has finalized (10–20 min per protein), affinity values or calculated binding energy were displayed as (-) kcal/mol. Each docking experiment was repeated twice (*n* = 2). If binding energy appeared as a positive value, it meant that binding of PGG to the target was energy-consuming, pointing to low affinity. Conversely, if binding energy was negative, PGG bound spontaneously to the target without energy consumption, releasing energy while binding. Therefore, the lower the binding energy of the docked PGG, the stronger the interactions with the target protein. In our studies, all PGG-target protein interactions had negative values (see Section 3). In addition, the software displayed the number and location of hydrophobic interactions and hydrogen bonds for each target-bound PGG simulation.

### 2.4. PGG Binding Patterns Analysis in Fresh Aorta

To mimic AAA, fresh, cannulated swine abdominal aorta segments (*n* = 3) were treated endoluminally with a mixture of elastase (10 U/mL) and collagenase (50 U/mL) in 100 mM Tris-HCl buffer and 1 mM CaCl2 in saline (pH 8) via infusion through the lumen, followed by capping the 2 Luer adapters. After incubation for 5 min, the arteries were rinsed in saline and then treated by endoluminal infusion with a clinical PGG formulation for 3 min, to mimic the clinical application, followed by a saline rinse, as previously described [14]. The PGG solution contained 3.0 mg/mL PGG in buffer and contrast material (244 mg/mL iopamidol) buffered with bicarbonate to pH 7.1. Tissue samples were then stained en bloc with FeCl_3_ for 5 min, rinsed in saline, and photographed. Cryosections at 5 µm thickness were counterstained with Light Green and imaged. From histology, tissue-bound PGG appeared as black deposits on a green background.

### 2.5. Diffusion of PGG through Pure Fibrin Gels and Intra-Luminal Thrombus (ILT)

The experimental setup consisted of 5 mL borosilicate glass pipettes (VWR #93000–696), cut to 7 cm length using a glass tubing cutter, connected to a 3-way stopcock via a barbed Luer adaptor and a segment of 2 cm long silicone tubing. A 60 mL syringe without the plunger served as a reservoir filled with PBS. This was connected to the 3-way stopcock via a 25 cm long vertical silicone tube (for the 30-mmHg experiment) and a 120 cm long vertical silicone tube (for the 100-mmHg application).

The fibrin gels were prepared by mixing bovine plasma fibrinogen and bovine plasma thrombin at 4 mg/mL and 0.8 U/mL final concentrations, respectively, in 20 mM HEPES buffer, 150 mM NaCl, 5 mM CaCl_2_. The mixture was drawn into the cut glass pipettes, the tube ends were sealed with parafilm, placed vertically in a stand, and allowed to clot at 37 °C for 30 min. The parafilm was removed from the glass tubes just before utilization. The glass tube containing the fibrin gel was connected to the PBS reservoir placed at 25 cm or 120 cm height to simulate 30 mmHg and 100 mmHg, respectively. In pilot studies, migration of red food dye (FD&C Red #40, 50 µL) through the 5 cm long gel column was observed visually by taking time-stamped photographs every 3–4 min. The dye diffused at a rate of approximately 1.25 mm/min at 30 mmHg and at 10 mm/min at 100 mmHg. The 50 µL volume applied was calculated from a typical situation where 25 mL of PGG is applied to an AAA of 4.5 cm diameter, 70% covered with a thrombus. PGG solution (50 µL) spiked with red dye exhibited similar diffusion characteristics (not shown). To test PGG diffusion through pure fibrin gels, 50 µL of PGG solution (same 3.0 mg/mL buffered solution containing contrast material as described above) was loaded on top of the fibrin gel and followed by connecting the glass column to a PBS reservoir and allowing the PBS to flow through the column while eluent fractions were collected. Pressure was applied at 30 mmHg for 3 min (by placing the reservoir at 25 cm height), during which, 2 fractions of 100 µL each were collected. Then, pressure was increased to 100 mmHg (by placing the reservoir at 120 cm height) and 100 µL fractions collected every minute for up to 25 min. The 3 min, 30 mmHg application mimicked the FIH clinical scenario where pressure inside the AAA was reduced to approximately 30 mmHg during intraluminal infusion of PGG for 3 min, followed by exposure of the aneurysmal sac to normal blood pressure of approximately 100 mmHg. Experiments were repeated twice (*n* = 2). PGG content was assayed in each fraction using the Folin–Ciocalteu reagent as described earlier and values were calculated as % of initial applied amount (means +/− standard deviation) [11,14].

Deidentified leftover ILT tissue was obtained from a consenting AAA patient during an open surgical repair procedure performed at PRISMA Health Upstate, Vascular Surgery, Greenville, SC. Ethics approval was obtained from the Institutional Review Board (IRB) at PRISMA Health for the PRISMA Health Biorepository that collects the tissue samples for this and other studies ongoing at the center. The freshly collected ILT tissue was made available as part of a research agreement between Clemson University and the PRISMA Health Biorepository tissue bank. The patient signed a written informed consent to participate in the PRISMA Health Biorepository. The Biorepository is an IRB approved human tissue bank which provides deidentified human tissue specimens for a wide variety of research nationally and internationally. Patients consented to participate in the Biorepository separately from their procedural consent. As a participant, the patient was informed that the tissue being collected was routinely disposed of unless being included in the Biorepository. The tissue was of no value to the patient, its collection did not pose any additional medical or privacy risks, and through inclusion in the Biorepository, the tissue would be used for further research. This was all reviewed and confirmed before the tissue was obtained.

The Biorepository staff obtained the tissue samples from the operating room, deidentified the sample, ensured the consent process was complete and then provided us with access to specimens in a blinded fashion. and after deidentification, was immediately transported to the Clemson lab. The authors of this study did not have access to information that could identify individual participants during or after data collection. Tissue collection for this study followed the recommendations outlined in the FDA OMB#0910-0582 “Guidance On Informed Consent For In Vitro Diagnostic Device Studies Using Leftover Human Specimens That Are Not Individually Identifiable”.

ILT core samples (*n* = 3 samples collected from different areas of the ILT), obtained within 2 h of collection, were used for PGG diffusion studies using a modified perfusion setup whereby the thrombus core sample was immobilized into a column adapted with a 0.5 µ sieve at the distal end. PGG solution (same 3.0 mg/mL buffered neutralized solution containing contrast material as described above) was applied on top of the thrombus, the system was connected to the perfusion setup and 30 mmHg of PBS column applied for 3 min to mimic the clinical PGG application, followed by 100 mmHg of PBS. Elution fractions were collected manually at the distal end of the ILT and analyzed for PGG content. PGG content was assayed in each fraction using the Folin–Ciocalteu reagent as described above for fibrin gels and PGG values expressed as % of initial applied amount (means +/− standard deviation). ILT samples were also fixed in formalin, processed for histology, and stained by H&E for overall morphology of the thrombus.

Statistical analysis: The results are presented as the mean +/− standard deviation. Statistical analysis was performed using Tukey’s student two tailed *t*-test in Microsoft Excel. Statistical significance was determined at a confidence interval of 95% (*p* < 0.05).

## 3. Results

### 3.1. Peptide Fingerprinting

**Figure 2:**
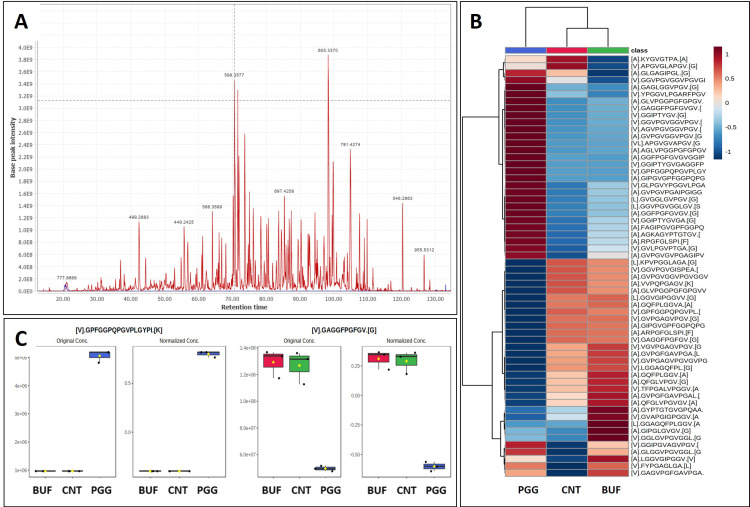
Peptide fingerprinting of human aortic elastin. (**A**) Representative chromatogram of elastin peptides obtained by elastase digestion of buffer-treated elastin control (BUF). (**B**) Heat map of the abundance of elastin peptides across PGG-treated elastin (PGG), vehicle control buffer with contrast (CNT) and BUF groups. Dark red, highest abundance, light blue, lowest abundance. (**C**) (**Left**) higher abundance of peptide [V].GPFGGPQPGVPLGYPI.[K] in PGG-treated elastin, (**right**) lower abundance of [V].GAGGFPGFGV.[G] elastin peptide after PGG treatment (PGG) as compared to buffer control (BUF) and vehicle control with contrast (CNT).

**Figure 3:**
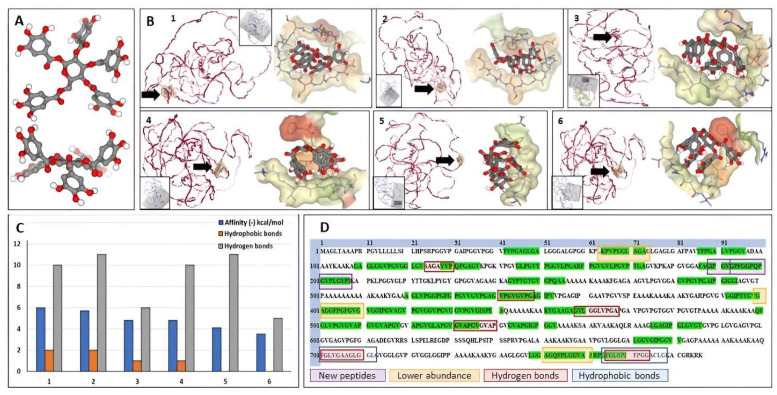
Docking of PGG onto elastin and localization of molecular interactions. (**A**) Three-dimensional structure of the PGG molecule, top view, and below, side view. Red balls depict the oxygen moieties. (**B**) Docking results from the study on PGG interactions with elastin (ELN). Interactions were mapped in six different ELN protein areas (labeled 1 to 6) by changing the interaction box coordinates (gray box in each insert), covering the entire protein structure. In each figure (1 to 6), the ELN protein is shown in red on the left, and a black arrow highlights the PGG binding site; on the right, a close-up of the PGG molecule interacting with the elastin chain. Note that PGG can acquire multiple flexible configurations. (**C**) Docking results show affinity values (binding energy) of PGG binding to the different areas in the ELN protein, expressed as (-) kcal/mol (blue bars), the number of hydrophobic bonds (orange bars), and hydrogen bonds (gray bars). (**D**) Amino acid structure of the canonical human ELN protein and mapping of salient PGG interactions. Green highlights depict the elastase-generated peptide sequences as determined by LC/MS. Purple squares depict the two new elastase-generated peptides that appeared only after PGG treatment, and the orange squares, the three peptides that exhibited lower abundance after PGG treatment of elastin. Overlapping the peptide fingerprinting data with the docking data revealed six areas of hydrogen bonding between PGG and elastin (red squares) and two main areas of hydrophobic bonding between PGG and ELN (blue squares).

In the first study, we treated pure human aortic elastin with PGG and analyzed the type and abundance of elastin peptides. Results were compared to elastin samples treated with the vehicle control (buffer and contrast material) and the vehicle without contrast material (to assess the effects of contrast material). Overall, the digestion of elastin treated with vehicle generated 58 peptides, covering about 50% of the elastin sequence (Figure 2A). By comparison, PGG-treated elastin generated 60 peptides, of which 2 new peptides had appeared in addition to the 58 peptides characteristic of our digestion conditions. These peptides were: [A].GIPGVGPFGGPQPGVPLGYPI.[K] localized at amino acids 188–208 and [V].GPFGGPQPGVPLGYPI.[K] at amino acids 192–208 in the elastin sequence (Figure 3D). When comparing the abundance of elastin peptides in the three groups, the BUF and CNT controls were very similar, suggesting that contrast material does not inhibit elastase and only slightly changes peptide patterns after elastase. PGG-treated elastin patterns were distinct from BUF and CNT controls, as evidenced by the heat map representation (Figure 2B), where PGG samples exhibited significantly altered abundance. About half of the peptides obtained from PGG-treated elastin were generated at higher abundance and about half at lower abundance. Comparing individual peptides several peptides were found in lower abundance in PGG treated elastin: [L].KPVPGGLAGA.[G] (63–74), [V].GAGGFPGFGV.[G] (398–410) and [A].GQFPLGGVA.[A] (750–761) (Figure 2C). These peptides were also mapped to the amino acid composition of the canonical human elastin (Figure 3D).

### 3.2. Molecular Docking of PGG onto Elastin

To better understand the interactions between PGG and elastin, we performed docking simulations using SeamDock as a web interface and the canonical human elastin structure. In water, PGG appears as a relatively planar molecule with the five galloyl residues pointing away from the center of the molecule and away from each other, similar to a “fan blade” (Figure 3A). Elastin appears as a random coil structure with very few alpha-helices in its structure. Molecular docking of PGG onto elastin in different areas of the molecule (Figure 3B), covering the entire elastin structure, showed that PGG binds preferentially to six areas with different affinities, as shown by the binding energy ranging from −3.5 to −6 kcal/mol and sustained by 0 to 2 hydrophobic bonds and 5 to 11 hydrogen bonds. These bonds were also mapped to the amino acid composition of the canonical human elastin (Figure 3D). Hydrophobic bonds between PGG and elastin appeared mostly towards the C-terminal of the elastin molecule (amino acids 700–712 and 764–778). Hydrogen bonds between PGG and elastin were localized in the same C-terminal area (701–710, 766–744) and throughout the elastin molecule (124–130, 341–348, 458–467, 531–540). Analysis of the docking images showed that PGG could acquire multiple flexible configurations to fit within the binding “pocket”, either spread out with all galloyl residues establishing bonds in multiple directions or a “clenched fist” configuration, with 3–4 galloyl residues concentrated in one single plane. Docking results (Figure 3C) show the binding energy of PGG to the different areas in the ELN protein, the number of hydrophobic bonds (orange bars), and hydrogen bonds (gray bars). Figure 3D depicts the salient features of PGG binding to human elastin resulting from overlapping the peptide fingerprinting data with the docking simulations.

### 3.3. Molecular Docking of PGG onto Other Normal Vascular Extracellular Matrix Proteins

**Figure 4:**
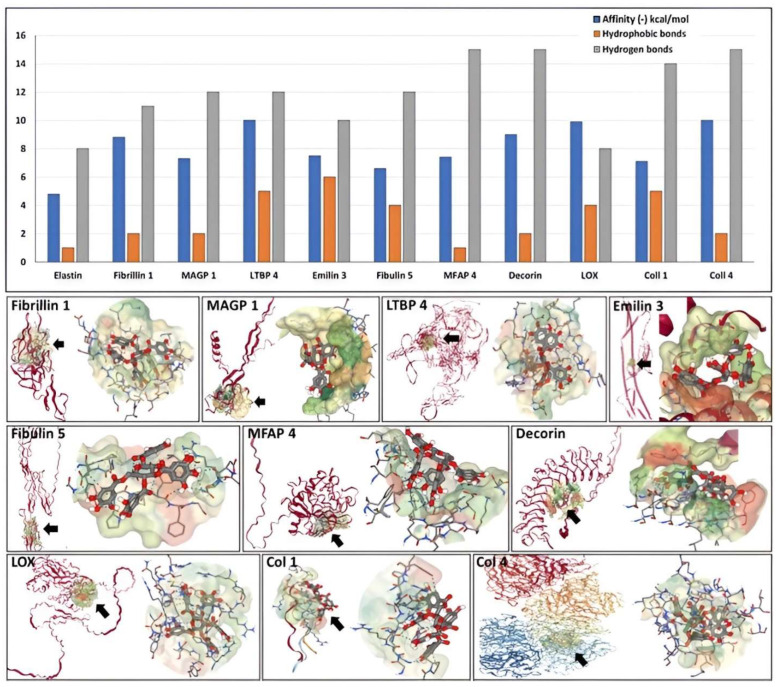
(**Top**) Affinity of PGG to the different components of the aortic wall depicted as binding energy values expressed as (-) kcal/mol (blue bars), the number of hydrophobic bonds (orange bars), and hydrogen bonds (gray bars) established between PGG and the proteins. (**Bottom**) Docking of PGG onto the major components of the aortic wall. The target protein (ribbon format) is shown in red on the left in each figure. A black arrow points to the most favorable calculated PGG binding site; on the right, a close-up of the PGG molecule interacting with the protein chain within the “pocket”. Note that PGG can acquire multiple flexible configurations.

To complement the data on elastin, we evaluated the potential of PGG to bind to the major fibrillar components of the elastic fiber and collagen using docking simulations. Table 1 provides a summary of the proteins analyzed and docking parameters. PGG exhibited a high affinity towards all proteins tested, including fibrillin 1 (binding energy of −9 kcal/mol), which makes up most of the elastin-associated microfibrillar “sheath” covering elastic fibers. A very high affinity of PGG was found towards MFAP 4 (−15 kcal/mol), followed by fibulin 5, LTBP 4, and MAGP 1 (−12 kcal/mol), followed by EMILIN 3 (−10 kcal/mol) and LOX (−8 kcal/mol). These high affinities were sustained by multiple hydrophobic bonds and hydrogen bonds (Figure 4). PGG was also predicted to bind strongly to the collagen type I triple-helical repetitive sequence and to the collagen type IV non-collagenous (NC) 1 domain (−7 and −10 kcal/mol, respectively) and to the decorin proteoglycan core protein (−9 kcal/mol), which is closely associated with collagen fibers. These simulations show that PGG has the ability to bind to single protein chains (intramolecular) or to “pockets” created by multiple chains (intermolecular).

### 3.4. Molecular Docking of PGG onto Proteins Present in the Aneurysmal Aorta

**Figure 5:**
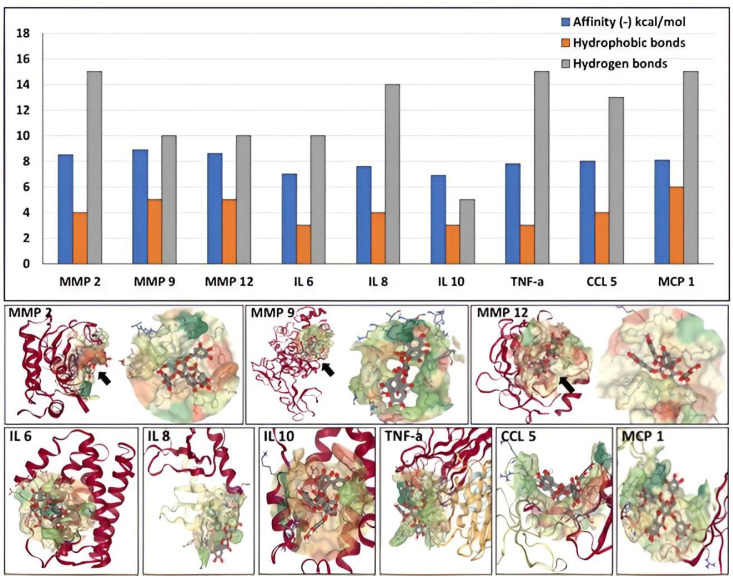
Affinity of PGG to MMPs and cytokines involved with AAA development and progression. (**Top**) Binding energy values are expressed as (-) kcal/mol, blue bars, the number of hydrophobic bonds (orange bars), and hydrogen bonds (gray bars) established between PGG and the proteins. (**Bottom**) Docking of PGG onto MMPs and cytokines. A black arrow points to the most favorable calculated PGG binding site; on the right, a close-up of the PGG molecule interacting with the protein chain within the “pocket”.

Next, we investigated the potential of PGG to bind to several components associated with the development and progression of AAA, namely active MMPs and cytokines, using docking simulations. PGG was predicted to bind with high affinity to the catalytic site of MMP 2, MMP 9, and MMP 12 (−8.2, −8.5 and −8.3 kcal/mol, respectively), sustained by multiple hydrophobic bonds and hydrogen bonds (Figure 5). PGG also exhibited significant affinity towards IL 6, IL 8, IL 10, TNF-a, CCL 5, and MCP 1 (−7 to −8 kcal/mol) with the ability to bind to single protein chains or to pockets created by multiple chains (Figure 5).

### 3.5. Diffusion of PGG through Fibrin Gels and Intra-Luminal Thrombus

**Figure 6:**
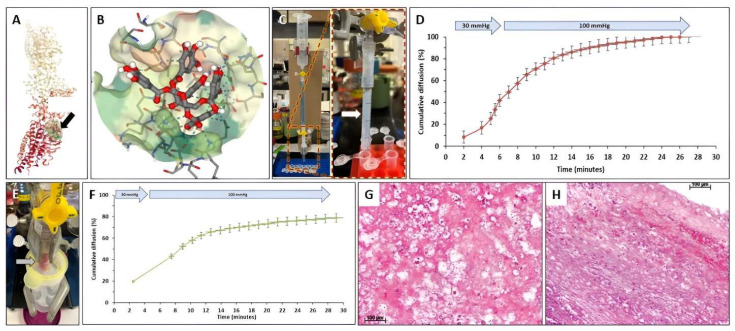
Interactions of PGG with fibrin and intraluminal thrombus. (**A**) Docking simulation of PGG binding to fibrin (black arrow). (**B**) Binding of PGG to a pocket in the fibrin molecule. (**C**) Hydrostatic pressure setup for evaluating PGG diffusion through fibrin gels. Fibrin gels were cast in a small glass tube attached to a funnel placed at various heights to simulate 30 mmHg and 100 mmHg. ((**C**), **Right**) detailed image of the fibrin gel (white arrow). (**D**) Cumulative PGG diffusion kinetics through fibrin gels at 30 mmHg followed by 100 mmHg pressures. PGG content was assayed in each eluting fraction collected at the outflow and expressed as % of starting PGG content (*n* = 2). (**E**) Hydrostatic pressure setup for evaluating PGG diffusion through intraluminal thrombus (arrow). (**F**) Cumulative PGG diffusion kinetics through intraluminal thrombus at 30 mmHg followed by 100 mmHg pressures. PGG content was assayed in each eluting fraction collected at the outflow and expressed as % of starting PGG content (*n* = 3). (**G**,**H**) Histological evaluation of the ILT. Hematoxylin and eosin stain, bar, 100 µm. Interactions of PGG with fibrin and intraluminal thrombus.

SeamDock simulation of PGG binding to fibrin was performed as described above. PGG was predicted to bind to the D-dimer of crosslinked fibrin with high affinity (−8.5 kcal/mol), involving three hydrophobic bonds and 15 hydrogen bonds (Figure 6A,B). Despite this potential for binding, PGG was observed to diffuse relatively rapidly through 5 cm of pure fibrin gel columns at a perfusion pressure of 30 mmHg for 3 min, followed by 100 mmHg. Transit occurred within 20–30 min, with almost complete recovery in fractions collected in the outflow of the fibrin gel column (Figure 6C,D). Subsequent experiments utilizing a diffusion chamber assessed whether the presence of patient intra-luminal thrombus (ILT) poses a barrier to PGG diffusion at physiologic pressures. When PGG was applied to a 4 cm long column of human intra-luminal thrombus, it was found to diffuse rapidly through the thrombus under 30 mmHg, followed by rapid elution from the ILT by PBS at 100 mmHg (Figure 6E,F). Quantification of PGG in the ILT after the diffusion study showed that about 17–20% of applied PGG remained in the thrombus. (From a total of 150 µg PGG applied to the column, about 120 µg PGG were transported through the ILT within 30 min.) Histology analysis of the ILT sample showed the presence of multiple pores of various diameters (10–100 µm) (Figure 6G,H).

## 4. Discussion

In our studies, PGG was predicted to bind to all normal vascular matrix components tested, such as elastin, elastin-associated microfibrils, collagens, and proteoglycan core proteins. The binding affinities were in the same range as those reported before for other proteins. Binding of PGG to a variety of targets has been investigated before via molecular docking, including using a SeamDock [31]. For example, molecular docking of PGG to two prostaglandin receptors was performed to understand its gastroprotective effects [33]. PGG was docked to the B-cell lymphoma 2 (bcl-2) family of anti-apoptotic targets (Bcl-2, BCL-XL, caspase 3, and caspase 9) with binding energies of −8.6, −7, −7.5, and 4.4 kcal/mol, respectively [34]. Additionally, PGG was shown via molecular docking to interact with vascular endothelial growth factor (VEGF) signaling molecules, VEGF-A,VEGF receptor (VEGFR-2), protein kinase C (PKC), rapidly accelerated fibrosarcoma (RAF), mitogen activated protein kinase (MEK), extracellular signal regulated kinase (ERK), and protein kinase B (AKT) with binding affinities of −7.9, −8.3, −8.6, −3.7, 10.1, −9, and −10.8 kcal/mol, respectively [35]. Recently, PGG was found to bind to the spike receptor binding domain of SARS-CoV-2 through molecular docking, with a −8 kcal/mol binding energy [36]. Furthermore, in our work, PGG was projected to bind to the active site of three major MMPs and to several cytokines, essential elements associated with the development and progression of AAAs.

PGG binding to pure human elastin is expected to occur at several sites with high affinity. PGG binding to elastin altered its cleavage pattern via elastase, potentially suggesting a mechanism of elastin stabilization, alluded to in earlier work [9,13,14,35,37,38,39,40,41,42,43,44]. Based on peptide fingerprinting and the molecular docking experiments, the C-terminal area of the elastin molecule around amino acids 700–770 appears to be a region of high affinity binding for PGG, with significant effects on elastase digestion patterns.

PGG binding to matrix proteins typically spans 8–9 amino acids, sometimes involving intramolecular interactions with a single linear protein chain or intermolecular bonds within “pockets” created by multiple neighboring chains. With alpha-helical proteins such as collagen, PGG tends to bind on the outside of the structures in a “clenched fist” configuration, surrounding or “wrapping” the fibrils like a glove. PGG binding to matrix proteins occurs mainly through multiple hydrophobic and hydrogen bonds. Hydrophobic bonds are typically established with Val and Leu in elastin and with Glu, Phe, Pro, Gln, and Arg in collagen, while hydrogen bonds are formed with Leu, Ala, and Gly in elastin and Gly, Gln, Arg, Pro in collagen. PGG established hydrophobic bonds with other proteins with Thr, Pro, Val, Tyr, Leu, Phe, His, Ala, Ile, and Trp.

In this study, PGG was also predicted to bind to catalytic sites of MMPs with high affinity, suggesting that PGG may act as a direct MMP inhibitor, as has been suggested previously [11,45,46]. PGG was also predicted to react with some of the cytokines involved in AAAs, potentially inactivating them. This would result in reducing the inflammatory burden typical of AAA, possibly reducing inflammatory cell recruitment, and potentially facilitating tissue healing. Overall, treatment of AAA tissues with PGG could potentially stabilize the existing matrix components and reduce their enzymatic degradation. As the peptide fragments generated during enzymatic degradation (matrix degradation products or “matrikines”) are typically pro-inflammatory, protection from further degradation, in turn, could further diminish inflammation. These actions might be responsible for the positive effects of PGG delivery to the aneurysmal aorta in rodents and swine. In these studies, delivery of PGG intra-luminally or peri-adventitially reduced aneurysmal expansion over time, sometimes leading to healing and reversal of the disease.

In our work, PGG was predicted to bind to crosslinked fibrin with high affinity but to diffuse rapidly through pure fibrin gels and human ILT under physiologic pressures, potentially because of the highly porous nature of the fibrin network. In our testing conditions, about 80% of PGG diffused freely through ILT, suggesting that ILT does not pose a barrier to PGG diffusion under physiologic pressures.

This study has several limitations: (1) One caveat of docking is that only one ligand molecule can be simulated at a time; multiple PGG molecules would likely bind to large molecules such as elastin or collagen. (2) The list of targets was not exhaustive but representative of our hypothesis. More studies are needed to cover the entire range of matrix components, enzymes, and multiple inflammatory cytokines to understand better the effects of PGG on AAA development and progression. It is also important to be able to validate results of molecular docking simulation with evidence from in vivo binding studies. (3) In the aneurysmal wall, there are probably multiple forms of degraded matrix proteins (elastin, EAMFs, collagen, etc.), cell debris, and infiltrated cells. We need to find out how PGG interacts with these components as well as with normal intact proteins. (4) We only had access to one ILT specimen; since ILT is reported to be heterogenous in nature and composition, more samples are needed to reach definitive conclusions.

## 5. Conclusions

PGG binds to the amorphous elastin core and the elastin-associated proteins, potentially protecting the entire macromolecular elastic fibril structure from degradation. Similarly, PGG binds to the collagen protein, and to the proteoglycans that decorate and protect the fibers from degradation. PGG also binds to MMPs and cytokines known to be present in AAAs, potentially exerting anti-inflammatory activities. These observations could potentially explain the observed tissue stabilization effect of PGG against the action of MMPs, and the “healing” effects of PGG on AAA development and progression. Finally, the patient ILT does not pose a barrier to PGG diffusion at physiologic pressures, opening opportunities for a deeper understanding of the clinical potential of PGG as a non-surgical treatment of AAA.

## Figures and Tables

**Table 1:** Target Proteins (Human).

Traditional Abbreviation	Protein Full Name	Classification and Properties	ID Code or Accession Number	Interaction Box Coordinates
NORMAL COMPONENTS				
ELN	Elastin	Matrix protein	* UniProt P15502; ** AlphaFold AF-P15502	6 separate areas
FLN 1	Fibrillin 1	Elastin-associated microfibrillar protein	*** RCSB 2W86; UniProt P35555	Whole molecule
MAGP 1 (MFAP 2)	Microfibril-associated glycoprotein 1	Elastin-associated microfibrillar protein	UniProt P55001; AlphaFold AF- P55001	Whole molecule
LTBP 4	Latent-transforming growth factor beta-binding protein 4	Elastin-associated microfibrillar protein	UniProt Q8N2S1; AlphaFold AF- Q8N2S1	Central core
EMILIN 3	Emilin 3	Elastin-associated microfibrillar protein	UniProt Q9NT22; AlphaFold AF- Q9NT22	Central core
FBLN 5	Fibulin 5	Elastin-associated microfibrillar protein	UniProt Q9UBX5; AlphaFold AF- Q9UBX5	Central core
MFAP 4	Microfibril-associated glycoprotein 4	Elastin-associated microfibrillar protein	UniProt P55083; AlphaFold AF-P55083	Central core
DCN	Decorin	Proteoglycan core protein	UniProt P07585; AlphaFold AF-P07585	Whole molecule
LOX 1	Protein-lysine Oxidase	Crosslinking enzyme	UniProt P28300; AlphaFold AF-P28300	Central core
COL 1	Collagen type I	Matrix protein; Fibrillar collagen	RCSB 7CWK	Central core (repetitive sequence)
COL 4	Collagen type IV, NC1 region	Basement membrane collagen	RCSB 1M3D	Central core
PATHOLOGIC COMPONENTS				
MMP 2	Matrix metalloproteinase 2	Enzyme involved in matrix degradation	RCSB 1QIB	Catalytic site (whole molecule)
MMP 9	Matrix metalloproteinase 9	Enzyme involved in matrix degradation	RCSB 1L6J	Catalytic site (whole molecule)
MMP 12	Matrix metalloproteinase 2	Enzyme involved in matrix degradation	RCSB 1JK3	Catalytic site (whole molecule)
IL 6	Interleukin-6	Cytokine	RCSB 1ALU	Whole molecule
IL 8	Interleukin-8	Cytokine	RCSB 1IL8	Whole molecule
IL 10	Interleukin-10	Cytokine	RCSB 2H24	Whole molecule
TNF-α	Tumor necrosis factor alpha	Cytokine	RCSB 1TNF	Whole molecule
CCL 5	CC chemokine 5	Cytokine	RCSB 5COY	Whole molecule
MCP 1	Monocyte chemoattractant protein 1	Cytokine	RCSB 1DOK	
Fibrin	D-dimer from cross-linked fibrin	Coagulation factor	RCSB 1N86	Whole molecule

* UniProt, uniprot.org; ** AlphaFold, alphafold.ebi.ac.uk; *** RCSB, rcsb.org.

## Data Availability

All relevant data are available within the manuscript.
